# Diagnosis and History Taking in Children with Autism Spectrum Disorder: Dealing with the Challenges

**DOI:** 10.3389/fped.2015.00055

**Published:** 2015-06-10

**Authors:** Michele Y. F. Kong

**Affiliations:** ^1^Department of Pediatrics, University of Alabama at Birmingham, Birmingham, AL, USA

**Keywords:** autism, non-verbal behavior, children, critical illness, communication

Autism spectrum disorder (ASD) has become one of the most common neurodevelopmental diagnoses, occurring in approximately 1 in 68 children in the United States (1). Physicians caring for children with autism during the child’s acute illness may face challenges in diagnosis and treatment that can negatively impact quality of care. The difficulties stem in large measure from the core features of the syndrome. Specifically, ASD is characterized by impairments in communication skills; impairments in reciprocal social interactions; and restricted, repetitive, and stereotyped patterns of behavior and interests (2, 3). In addition to these core diagnostic features, ASD is often accompanied by impairments in cognitive and adaptive functioning, attention deficit, sensory processing disorder, and aggression or self-injurious behavior (4). In the presence of an acute illness, it can be extremely difficult to disentangle the core behaviors of autism from other illnesses. This challenge is intensified in a subgroup of these children who have very little or no expressive language (often characterized as “non-verbal” or “minimally verbal”) (5).

As a case in point, let us consider a non-verbal child who presented to the emergency room (ER) with a 1-week history of fever, vomiting, and poor oral intake. He was generally a happy child, although in the last several weeks he had begun to have periods of agitation that manifested as inconsolable crying and self-injurious behaviors. The father also noted that he seemed to be “wobbly.” The physician on duty evaluated him, and a basic workup revealed normal electrolytes and serum glucose level. An abdominal KUB was obtained and was unremarkable. On exam, the physician noted a child who was agitated and had periods of head hitting. His vital signs were significant for tachycardia but were otherwise within normal limits. The child was discharged home with a diagnosis of “viral gastroenteritis” and the family was told to provide symptomatic care (antipyretics and hydration) and to have a follow-up appointment with the pediatrician.

Let us now consider this case from the physician’s viewpoint. Who among us would have made the same diagnosis? It was a typical day in the ER, and this was the 11th hour of the 12-h shift. We have seen many patients in the last several hours, most of whom presented with vomiting and diarrhea – this was, after all, the theme of the day: kids with the “GI” bug. The only abnormal vital sign was an elevated heart rate, but the child was febrile and agitated. He was wobbly, but his blood glucose was normal, and a reliable neurologic examination was impossible. He was agitated and was hitting his head, but was this not just his normal behavior, especially under a new and stressful environment?

Teasing out the core deficits of ASD from an acute underlying medical condition is a real and significant challenge for medical providers. It is important to realize that not all children with autism will engage in aggressive behaviors toward themselves (such as head hitting or biting), and presentation with such behaviors may not simply be manifestations of autism. We need to always consider if these noteworthy, abnormal behavioral findings could potentially be due to a more sinister cause, such as intense abdominal pain from a ruptured appendix, severe headache from an intracranial pathology, or pain secondary to fractures from an unwitnessed trauma. Irritability and social withdrawal are typical sick behavior in otherwise healthy children experiencing pain, discomfort, and anxiety. They are even more important clues in non-verbal patients, including many children with autism (6). To compound matters, new environments are often difficult for children with autism. They are extremely sensitive to environmental stimuli and are highly dependent on predictable routine. The bright lights, smell, and fast pace of the ER can easily overwhelm the senses of an autistic child. Furthermore, the wait time is often long, and in a crowded and loud room. The barrage of questioning and interactions with multiple care providers, ranging from ancillary staff to nurses and physicians can be difficult for them to process. Unfamiliar settings or procedures will lead to anxiety, which often manifests as disruptive or aggressive behaviors because of limitations in social and communication skills. In addition, because of cognitive impairments and an inability to understand what is being expected of them, children with autism may become frustrated. This combination of sensory overload, fear, anxiety, and frustration can lead to exacerbation of behavior and a vicious cycle of confusion for the patient and caregiver alike. So, the very setting in which the physician sees the child can often contribute to an increase in symptoms, all of which can complicate the evaluation of the presenting illness seen in an ER.

In a recent study, healthcare disparities including worse patient-provider communication were found between non-autistic and autistic adults (7). In addition, a survey of primary care physicians revealed that many medical providers are uncomfortable with their level of training in dealing with autistic adults (8). Although the data are scant in the pediatric population, most of us who have encountered and managed an autistic child would concur that the challenges are the same, if not magnified in young autistic children, and especially for those who are non-verbal.

As ASD has received increasing recognition, many developments have been made in the field, including advances in neuroimaging, genetics, and early behavioral interventions (9). But what techniques can we use in the ER to facilitate our communication with these children? Based on feedback from families and older, verbal autistic children, it is essential that the physician should speak to the child in a calm and simple manner. It is also critical to break instructions into simpler understandable steps. In addition, the language used should not just be simple but specific and concrete. Concrete language results when our words convey the literal meaning of the words spoken. For example, a child with autism will be more likely to understand “Get up onto the table” rather than “Why don’t you hop up here and let me check you out.” Children with autism may not understand sarcasm or irony and shouldn’t be expected to “read between the lines.” It is imperative that we stay away from using metaphors and synonyms or making analogies. For example, a sentence such as “You see, screaming isn’t helping much, is it?” is better said as “Please be quiet.” Another common problem is giving instructions in the form of questions. “Are you coming over here?” can be difficult to comprehend compared to “Come here.” Finally, after giving a simple, concrete instruction, it is important to allow a child with autism adequate time to respond. Often, it may appear that the child did not understand the question (prompting a barrage of repeated questions from the adult), but all that is needed is additional time for the child to process the incoming information. Repeating the question to force a response, or constant talking, can lead to worsening of challenging behaviors as the child becomes overwhelmed with the verbal information. These simple techniques can easily be applied in our encounters with an autistic child, and they can result in dramatic improvements in our history taking and communication.

But more can, and should be done. When caring for patients with limited verbal communication, it is vital that we shift our focus from language to *behavior*. Careful attention to behavior is the first step toward achieving accurate diagnoses. In the case described above, the child progressively worsened over the night and was brought back to the ER within 12 h, now with altered mental status and progressive shock. He was intubated and admitted to the Intensive Care Unit (ICU), where further evaluation revealed bacterial meningitis. A detailed history from the father revealed that the aggression and head hitting were a new behavior for his son. Over several days preceding his initial presentation in the ER, the intensity and frequency of the behavior had increased. Finally, in the week before admission, he had also begun to have intense periods of vomiting and persistent fever. The head hitting was a critical piece of his history. It was a key clue that was missed – a clue that was a *new symptom* suggesting a headache. Instead, this critical symptom of meningitis was overlooked as merely normal autistic behavior. Taking a detailed history of a child’s typical behaviors enables the physician to rightly interpret new symptoms to create a differential diagnosis. Taken together with his other presenting complaints (fever and vomiting) headache would have suggested the possibility of a central nervous system infection, and evaluation to rule out meningitis would have ensued. In the absence of verbal information from the child, it is even more vital than usual to know how to “read” behavioral cues.

For many children with chronic conditions, including those with ASD, medical care is fragmented, with no single provider providing oversight. It is not uncommon for these families to seek consultation from several physicians during the same period, and for the same constellation of symptoms related to their underlying condition. This reality can make it very challenging for the frontline physician to obtain a comprehensive past medical history. Families are often sleep deprived, overwhelmed, and may also be agitated due to the level of demands surrounding the presenting illness. In such a highly stressful situation, they may not offer key information unless specifically asked (“Is the head hitting a new behavior” versus the assumption that it was typical). Questions addressing behavior should be asked early and should include such queries as these: is this a *new* behavior? Is the behavior a *change* from the typical ASD presentation for the child? Is the behavior an escalation of existing behavior? What are the typical triggers and consequences of the behavior? And, finally, how is the behavior eliminated if it is not new? Answers to these questions will guide the physician in additional history taking and management of the acute illness at hand.

It is incumbent upon us to have a high index of suspicion for potentially life-threatening illnesses in all our patients, and especially in vulnerable populations. For instance, because the clinical exam alone is unable to reliably predict serious illness in neonates with fever, often the clinician will initiate a septic workup. Likewise, in autistic children with communication impairments, it is imperative that *new* and *different* behaviors are not ignored, and it is wise to presume that there is an underlying medical cause for them until proven otherwise.

In this case, the child survived his illness but suffered significant morbidity, including the development of hydrocephalus requiring a ventricular shunt placement and sadly, paraplegia of his lower extremities (from septic emboli involving the spinal cord).

The take home point? Ask, and ask some more. Never be content with making assumptions without strong evidence. As frontline physicians in the ER and ICU, we treat the sickest patients. But, paradoxically, it is often the ones who do not present *in extremis* – whose illnesses can be explained (or explained away) by a simple and straightforward diagnosis, who will challenge us as physicians the most. They are they ones who require us to be on our game, to be astute, and to pay attention to details. They are the ones who *require* us to be the better physician.

## Conflict of Interest Statement

The author declares that the research was conducted in the absence of any commercial or financial relationships that could be construed as a potential conflict of interest.
